# Electrocardiogram-gated cardiac computed tomography-based patient- and segment-specific cardiac motion estimation method in stereotactic arrhythmia radioablation for ventricular tachycardia

**DOI:** 10.1016/j.phro.2025.100700

**Published:** 2025-01-21

**Authors:** Jingyang Xie, Alicia S. Bicu, Melanie Grehn, Mustafa Kuru, Adrian Zaman, Xinyu Lu, Christian Janorschke, Luuk H.G. van der Pol, Martin F. Fast, Jens Fleckenstein, Marcus Both, Stephan Hohmann, Egor Borzov, Peter Winkler, Roland R. Tilz, Dirk Rades, Frank A. Giordano, Daniel Buergy, Boris Rudic, David Duncker, Roland Merten, Tomer Charas, Mahmoud Suleiman, Thomas Brunner, Daniel Scherr, Evgeny Lian, Achim Schweikard, Oliver Blanck, Judit Boda-Heggemann, Lena Kaestner

**Affiliations:** a Institute of Robotics and Cognitive Systems, University of Luebeck, Luebeck, Germany; b Department of Radiation Oncology, University Medicine Mannheim, Medical Faculty Mannheim, Heidelberg University, Mannheim, Germany; c DKFZ Hector Cancer Institute, University Medical Center Mannheim, Germany; d Department of Radiation Oncology, University Medical Center Schleswig-Holstein, Kiel, Germany; e Department of Radiology, University Medicine Mannheim, Medical Faculty Mannheim, Heidelberg University, Mannheim, Germany; f Department of Internal Medicine III (Cardiology, Angiology, and Internal Intensive Care Medicine), University Medical Center Schleswig-Holstein, Kiel, Germany; g Department of Radiotherapy, University Medical Center Utrecht, Utrecht, the Netherlands; h Department of Radiology and Neuroradiology, University Medical Center Schleswig-Holstein, Kiel, Germany; i Hannover Heart Rhythm Center, Department of Cardiology and Angiology, Hannover Medical School, Hannover, Germany; j Cardiology & Internal Intensive Care Medicine, St. Bernward Hospital, Hildesheim, Germany; k Department of Radiotherapy, Division of Oncology, Rambam Health Care Campus, Haifa, Israel; l Department of Therapeutic Radiology and Oncology, Medical University of Graz, Graz, Austria; m Department of Rhythmology, University Heart Center Luebeck, University Hospital Schleswig-Holstein, Luebeck, Germany; n German Center for Cardiovascular Research (DZHK), Partner Site Hamburg/Kiel/Luebeck, Germany; o Department of Radiation Oncology, University of Luebeck, Luebeck, Germany; p Department of Internal Medicine I, Section for Electrophysiology and Rhythmology, University Medicine Mannheim, Medical Faculty Mannheim, Heidelberg University, Mannheim, Germany; q German Center for Cardiovascular Research (DZHK), Partner Site Heidelberg, Mannheim, Germany; r Department of Radiotherapy, Hannover Medical School, Hannover, Germany; s Division of Pacing and Electrophysiology, Rambam Health Care Campus, Haifa, Israel; t Division of Cardiology, Medical University of Graz, Graz, Austria

**Keywords:** Ventricular tachycardia, Stereotactic arrhythmia radioablation, Cardiac motion estimation, AHA 17-segment model, ICD lead tip motion, Cardiac ITV margin estimation

## Abstract

**Background and purpose:**

Motion management strategies such as gating under breath-hold can reduce breathing-induced motion during stereotactic arrhythmia radioablation (STAR) for refractory ventricular tachycardia. However, heartbeat-induced motion is essential to define an appropriate cardiac internal target volume (ITV) margin. In this study, we introduce a patient- and segment-specific cardiac motion estimation method and cardiac motion data of the clinical target volume (CTV), ICD lead tips and left ventricle (LV) segments.

**Materials and methods:**

Data from 10 STAR-treated patients were retrospectively analyzed. The LV was semi-automatically segmented according to the 17-segment model. Electrocardiogram-gated contrast-enhanced breath-hold cardiac CTs were automatically non-rigidly registered for motion estimation. The correlation and significant differences between ICD tip motion and CTV motion were assessed using the Pearson correlation coefficient (PCC) and Wilcoxon signed-rank test, while spatial discrepancies with both CTV and segment motion were quantified using the Euclidean distance.

**Results:**

The CTVs (center of mass) moved 3.4 ± 1.4 mm and the ICD lead tips moved 4.9 ± 2.2 mm. The maximum motion per patient was observed in basal and mid-cavity LV segments in 3D. The PCC showed a strong positive motion correlation between the ICD tip and CTV in 3D (0.84), while the p-values indicated statistically significant differences in the right-left, anterior-posterior and 3D directions.

**Conclusion:**

The proposed methods enable patient- and segment-specific cardiac ITV margin estimation. The motion in most LV segments was limited, however, cardiac ITV margins may need adjustment in individual cases. The impact of cardiac motion on the dosimetry needs further investigation.

## 1 Introduction

Ventricular tachycardia (VT) is a severe life-threatening arrhythmia originating from the ventricles, potentially causing sudden cardiac death [1], [2]. Traditional management of VT includes antiarrhythmic drugs, catheter ablation and implantable cardioverter-defibrillator (ICD) [1], [3]. However, they have limitations such as pharmacological toxicities, recurrence of arrhythmias and potential complications [4], [5].

Recently, stereotactic arrhythmia radioablation (STAR) has emerged as a novel and non-invasive treatment for refractory VT [6], [7], [8], [9], gaining acceptance in the cardiology community [10]. STAR utilizes focused radiation beams to ablate arrhythmogenic heart tissue with a single fraction radiotherapy dose of 25 Gy [8], [11].

The precision of STAR relies on accurately identifying and targeting of the VT substrate in the left ventricle (LV), requiring knowledge of cardiac anatomy and its dynamic behavior due to cardiac and breathing motion. The clinical target volume (CTV) defines the three-dimensional (3D) VT substrate, determined by the treatment team using all available electrophysiologic and imaging data [12]. The internal target volume (ITV) represents the CTV with a margin for internal motion. Respiratory motion can be accurately managed with gating, deep inspiration breath-hold (DIBH) or robotic tracking [13], [14]. However, cardiac motion, particularly the movement of the CTV and the American Heart Association (AHA) 17 segments of the LV [15], presents a significant challenge in the precise definition of cardiac ITV. This movement can lead to misalignments, which may reduce treatment effectiveness and increase the risk of harm to nearby organs at risk via dose wash-out [16]. Therefore, estimating cardiac motion is essential for defining an appropriate cardiac ITV margin, thereby enhancing the effectiveness of STAR.

Several studies on cardiac motion analysis for STAR have been published in recent years [17], [18], [19], [20], [21], [22]. A STOPSTORM.eu consortium review showed that cardiorespiratory motion is patient-specific, highlighting the need for personalized motion management in STAR [16]. However, studies focusing on patient- and segment-specific cardiac motion analysis of the CTV (especially at the voxel-wise level) and on the correlation between ICD lead tip motion and CTV/LV segment motion in STAR-treated VT patients remain limited.

This study aimed to estimate cardiac motion for the CTV and the 17 LV segments using electrocardiogram (ECG-)gated contrast-enhanced breath-hold computed tomography (CT) with an intensity-based non-rigid automatic image registration method as proof-of-concept. ICD lead tip motion was also calculated for evaluation of accordance with the CTV and LV segments motion. This patient- and segment-specific cardiac motion estimation method could enhance STAR treatment planning including individualized cardiac ITV margin estimation and the selection of cardiac motion mitigation strategies for individual patients. It has potential for future clinical applications in STAR.

## 2 Materials and methods

### 2.1 Dataset preparation

Case data for 10 VT patients who underwent STAR based on the RAdiosurgery for VENtricular TAchycardia (RAVENTA) trial protocol (NCT03867747) [11], [23] either directly within the RAVENTA study in Germany [11] or within harmonized trials in Austria and Israel (Trial Number: 202225964) [24] and/or within the STOPSTORM registry [8] were retrospectively analyzed. All patients received a single dose of 25 Gy prescribed to the surrounding isodose surface, ensuring compliance with critical structure constraints as outlined in the RAVENTA trial protocol [11]. Each patient had one CTV, except for two patients who had two CTVs. Detailed patient characteristics are summarized in Table 1.

**Table 1:** Patient characteristics. Each patient had one CTV, with the exception of patients 2 and 9 with two CTVs. Abbreviations: BMI = body mass index; LVEF = left ventricular ejection fraction; CTV = clinical target volume; LV = left ventricle; CT = computed tomography. *Based on the American Heart Association 17-segment model.

Patient	Age (years)	Sex (m, f)	BMI (kg/m^2^)	Cardiac condition	LVEF (%)	Catheter ablation history	CTV (cm^3^)	Targeted (partial) LV segments*	CT pixel spacing (mm)	CT slice thickness (mm)	Volumetric difference of the epicardial LV between end-diastolic and end-systolic phases (cm^3^)
1	70	m	23.6	Ischemic cardiomyopathy	23	1	6.7	4	0.375	0.3	42.3
2	84	m	29.4	Ischemic cardiomyopathy	33	3	CTV 1: 11.4; CTV 2: 21.1	CTV 1: 4, 5, 11; CTV 2: 1, 7	0.389	0.4	51.2
3	61	m	39.8	Ischemic cardiomyopathy	35	2	42.8	1, 2, 7, 8	0.460	0.4	81.8
4	74	m	23.2	Non-ischemic cardiomyopathy Dilated cardiomyopathy, valvular defect (tricuspid, mitral)	35	0	8.3	6, 11, 12	0.463	0.5	71.2
5	62	m	28.4	Dilated cardiomyopathy, valvular defect (mitral), atrial fibrillation	35	3	11.5	2, 3, 9	0.479	1.0	133.4
6	61	m	26.1	Dilated cardiomyopathy, valvular defect (tricuspid, mitral)	46	3	4.4	6, 12	0.461	0.5	55.5
7	75	m	31.0	Dilated cardiomyopathy	25	5	83.3	1, 5, 6, 7, 11, 12	0.490	0.8	103.6
8	72	m	33.1	Ischemic cardiomyopathy	25–30	3	31.2	5, 6, 11, 12	0.422	0.6	38.2
9	64	m	19.9	Non-ischemic cardiomyopathy	32	2	CTV 1: 8.2; CTV 2: 17.6	CTV 1: 3, 4; CTV 2: 1, 6	0.336	0.3	103.1
10	59	m	26.5	Non-ischemic cardiomyopathy	25	2	44.4	1, 6, 7, 12	0.387	0.4	82.4

Contrast-enhanced breath-hold cardiac CT with retrospective ECG gating was acquired, with reconstructions of the end-diastolic and end-systolic phases included as part of treatment planning for STAR. Cardiac substructures, with special emphasis on LV myocardium and aorta, were contoured on the cardiac CT at the end-diastolic phase in the radiotherapy treatment planning system by experienced senior physicians. In cases where the LV myocardium structure was not available, the TotalSegmentator extension (version 2.4.0) [25], [26] within the open-source 3D Slicer software (version 5.6.2) [27] was used for LV myocardium structure segmentation. The LV myocardium structure was then divided into 17 segments according to the AHA 17-segment model [15], using the in-house software CARDIO-RT [28], [29]. The CTV was predefined based on the electroanatomical maps with geometry created in end-diastole during sinus or slow-paced rhythm as previously described [11]. The contrast-enhanced cardiac CT datasets were in DICOM format, and the cardiac substructures were exported in a DICOM-RT Structure Set (RTSS) file.

All patients had at least one ICD lead positioned in the right ventricle (RV) near segment 14, and 5 patients had an additional ICD lead positioned in the right atrium. The tip of the ICD lead in the RV was manually segmented in the cardiac CT at both end-diastolic and end-systolic phases using the 3D Slicer software and exported in an RTSS file.

### 2.2 Intensity-based non-rigid automatic image registration

**Fig. 1:**
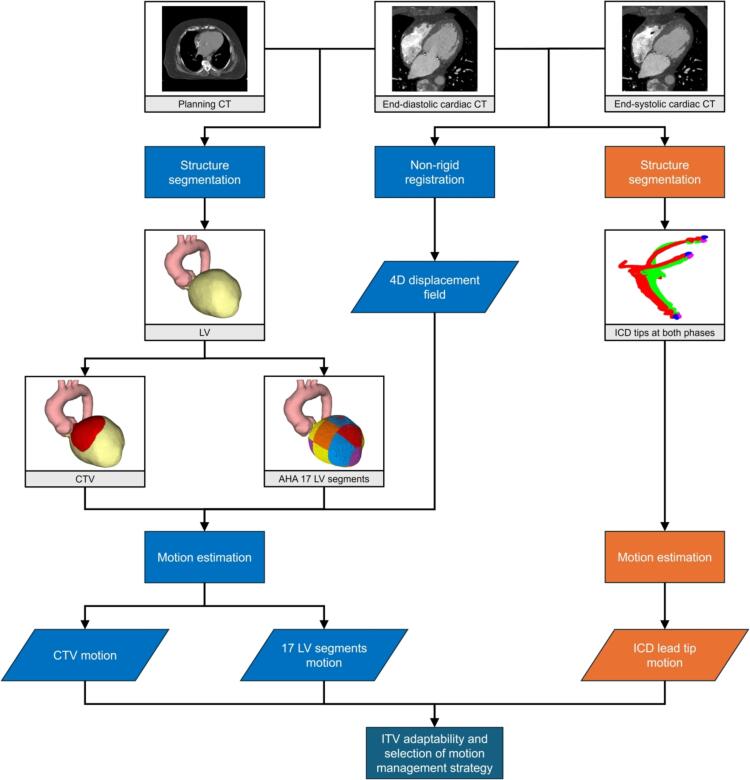
Diagram of cardiac motion estimation workflow. Processes and data in light blue boxes relate to the cardiac motion of the clinical target volume (CTV) and American Heart Association (AHA) 17 left ventricle (LV) segments, while those in orange relate to implantable cardioverter-defibrillator (ICD) lead tip motion. Abbreviations: CT = computed tomography; ITV = internal target volume. (For interpretation of the references to colour in this figure legend, the reader is referred to the web version of this article.)

**Fig. 2:**
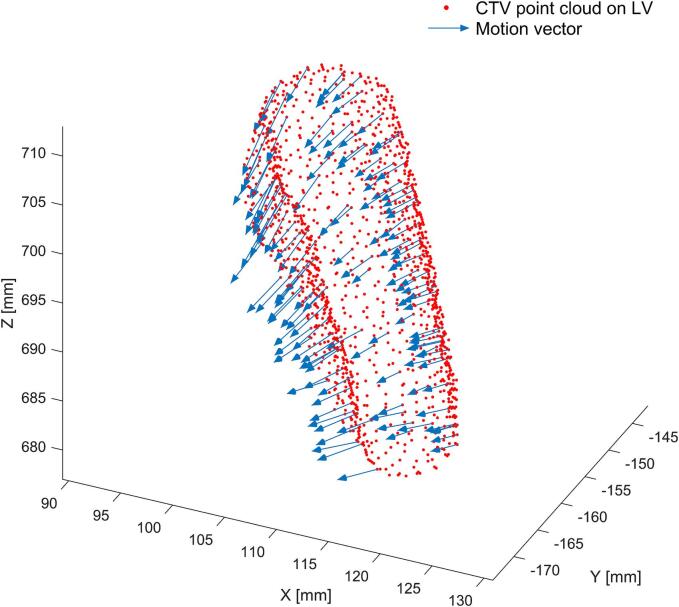
Motion vectors representing the deformation of a clinical target volume (CTV) point cloud on the left ventricle (LV) at the end-diastolic phase.

The 3D cardiac CT volumes at end-diastolic and end-systolic phases were registered using the MATLAB intensity-based non-rigid automatic image registration function (Image Processing Toolbox™, The MathWorks, Inc., Natick, Massachusetts, USA), resulting in a four-dimensional (4D) displacement field. For the image registration procedure, the cardiac CT volume at end-diastolic phase was displaced, while the cardiac CT volume at end-systolic phase was fixed. The output 4D displacement field contained displacements (unit: pixels) along the *x*-axis (right-left, RL), *y*-axis (anterior-posterior, AP) and *z*-axis (superior-inferior, SI). The displacements in millimeters can then be determined based on the CT pixel spacing and slice thickness and were treated as estimates of cardiac motion of the cardiac substructures within one cardiac cycle. Fig. 1 illustrates the cardiac motion estimation workflow. Fig. 2 shows the motion vectors representing the deformation of a CTV.

Afterwards, the image registration performance was evaluated using the mean squared error (MSE) and Pearson correlation coefficient (PCC, significance level = 0.05) of the Hounsfield unit (HU) for the cardiac CT voxels using MATLAB (version R2022a). A region of interest (ROI) was created in the cardiac CT volumes at both end-diastolic and end-systolic phases which includes the voxels of both the original and displaced LV myocardium structures. The MSE and PCC (*r*) between the original and displaced ROIs were calculated using the following formulas:

$$MSE=\frac{1}{N}\sum_{i=1}^{N}{\left(I_{original}\left(i\right)-I_{displaced}\left(i\right)\right)}^{2}$$

$$r=\frac{\sum_{i=1}^{N}\left(I_{original}\left(i\right)-{\overset{¯}{I}}_{original}\right)\left(I_{displaced}\left(i\right)-{\overset{¯}{I}}_{displaced}\right)}{\sqrt{{\sum_{i=1}^{N}\left(I_{original}\left(i\right)-{\overset{¯}{I}}_{original}\right)}^{2}}\sqrt{{\sum_{i=1}^{N}\left(I_{displaced}\left(i\right)-{\overset{¯}{I}}_{displaced}\right)}^{2}}}$$

where *I*_original_ and *I*_displaced_ are the intensities of the corresponding CT voxels in the original and displaced ROIs, respectively, *N* is the total number of voxels within the ROI.

### 2.3 Estimation of cardiac motion

After the image registration procedure, the output 4D displacement field was then applied to the points of the contours of CTV and 17 LV segments, resulting in displaced points of these cardiac substructures. The motion vector of the center of mass (CoM) of the CTV and 17 LV segments from the end-diastolic phase to end-systolic phase was calculated in the RL, AP, SI and 3D directions.

Since the motion of the CTV and the 17 LV segments is non-rigid due to heart contraction and expansion, the motion of the CoMs within the contoured structures does not fully represent their cardiac motion. Therefore, the motion of each point within the contoured cardiac substructures from the RTSS file was also calculated in the RL, AP, SI and 3D. Similarly, the ICD lead tip motion was also calculated for evaluation of accordance with CTV/LV segment motion. For patients with more than one ICD lead in the RV, only the most inferior one (near segment 14) was used for this evaluation.

### 2.4 Statistical analysis

Statistical analysis was performed using Microsoft Excel (version 2411) and R (version 4.2.1) within the RStudio environment (version 2022.07.2 + 576). The correlation and significant differences between ICD lead tip motion and CTV motion were assessed using the PCC (significance level = 0.05) and Wilcoxon signed-rank test (significance level = 0.05), while spatial discrepancies with both CTV and segment motion were quantified using the Euclidean distance. Values are given as mean ± standard deviation unless noted otherwise.

## 3 Results

### 3.1 Evaluation of image registration performance

After the image registration, for the ROI in the cardiac CT volumes, the MSE reduced by 40830 ± 15484 HU^2^ and the PCC increased from 0.81 ± 0.05 to 0.94 ± 0.02, indicating enhanced similarity between cardiac CT at end-systolic phase and registered cardiac CT at end-diastolic phase.

### 3.2 Cardiac motion estimation of CTV and 17 LV segments

**Fig. 3:**
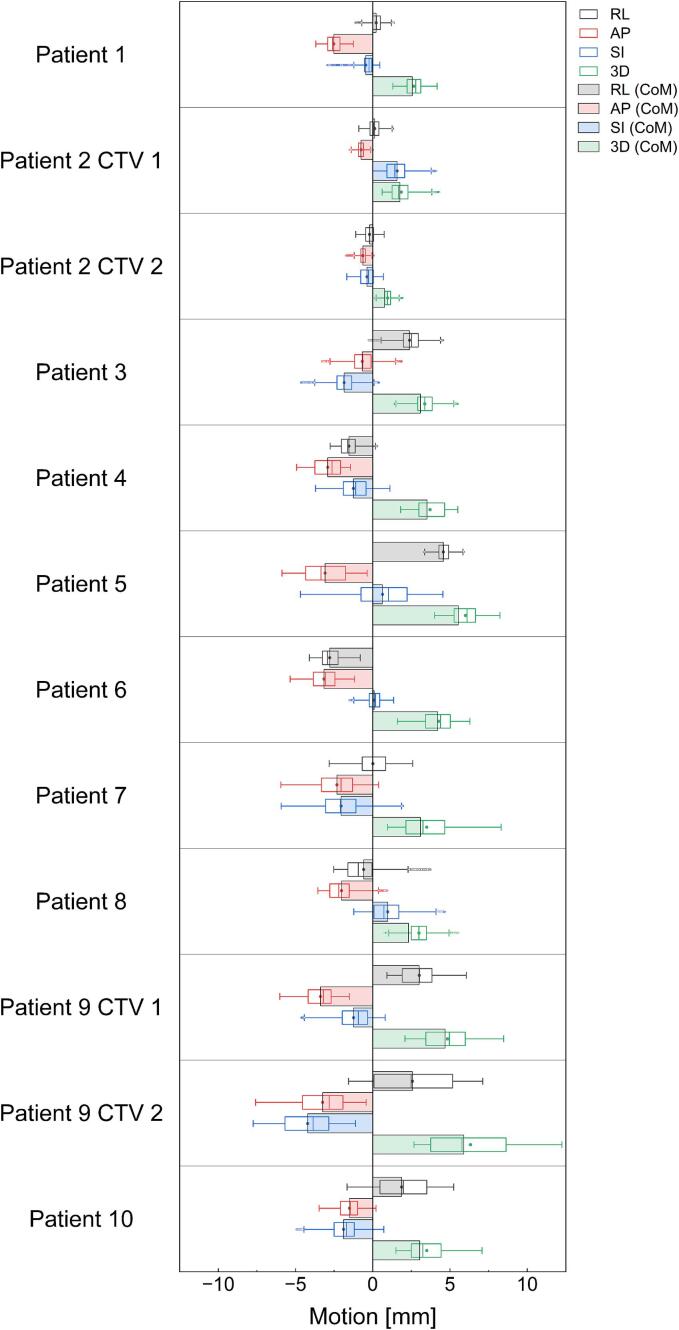
Cardiac motion of the center of mass (CoM) for individual clinical target volumes (CTVs, bar chart) and individual CTV points (box chart) in the right-left (RL), anterior-posterior (AP), superior-inferior (SI) and 3D directions. Each patient had one CTV, with the exception of patients 2 and 9 with two CTVs. Mean values are shown as dots in the box chart. For the cardiac motion of individual CTV points, the mean and median values are similar and comparable.

Fig. 3 presents the cardiac motion of the CoM for individual CTVs and individual CTV points in the RL, AP, SI and 3D directions (detailed numerical data are given in Supplementary Table S1). Comparing cardiac motion of the CoM for individual CTV with the mean value of the individual CTV points, the difference was 0.3 ± 0.2 mm. The absolute cardiac motion for the CoM was 1.7 ± 1.4 mm in the RL direction, 2.2 ± 1.0 mm in the AP direction, 1.4 ± 1.1 mm in the SI direction, and 3.4 ± 1.4 mm in 3D. Of the 12 CTVs (CoM), 10 exhibited cardiac motion < 5 mm in 3D.

Regarding the points of the CTVs, the mean cardiac motion in 3D was < 5 mm in 11 out of 12 CTVs; cardiac motion of CTV 2 from patient 9 was considerably larger, with a mean value of 6.6 mm.

**Fig. 4:**
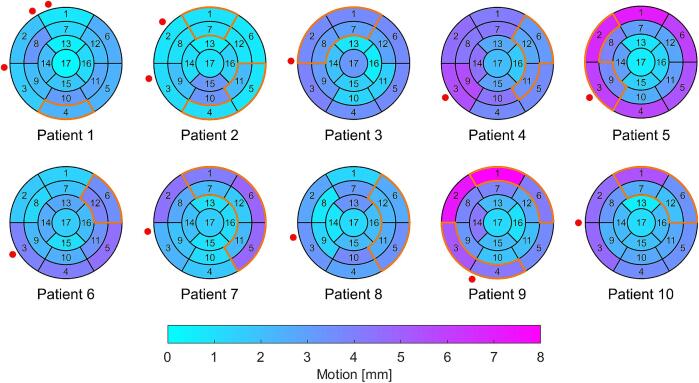
Cardiac motion of the center of mass for the American Heart Association 17 segments of the left ventricle in 3D on 17-segment bull’s-eye maps with the locations of the clinical target volumes (CTVs, orange borders) and implantable cardioverter-defibrillator lead tips (red dots) for each patient. Each patient had one CTV, with the exception of patients 2 and 9 with two CTVs. CTVs are numbered in ascending order from inferior to superior in the manuscript. (For interpretation of the references to colour in this figure legend, the reader is referred to the web version of this article.)

Among the 10 patients, cardiac motion across the 17 LV segments was < 5 mm in the RL, AP and SI directions, with the exception of patient 5 (segment 4 in the AP direction, segment 6 in the SI direction) and patient 9 (segment 2 in the AP direction, segment 1 in the SI direction). In 3D, cardiac motion of most of the segments was < 5 mm, except patient 4 (segments 3 and 9), patient 5 (segments 1, 2, 4, 5 and 6) and patient 9 (segments 1, 2 and 3; Fig. 4). The maximum motion per patient was observed in basal (segments 1, 3, 5 and 6) and mid-cavity (segments 8, 9 and 10) regions.

### 3.3 ICD lead tip motion estimation and its accordance with cardiac motion

The motion of the RV ICD lead tips (near segment 14) in the RL, AP, SI and 3D directions for the 10 patients can be found in Supplementary Table S1. The absolute motion of the ICD lead tips was 3.5 ± 2.8 mm in the RL, 1.5 ± 1.2 mm in the AP, 1.9 ± 1.2 mm in the SI and 4.9 ± 2.2 mm in 3D. Comparing the ICD lead tip motion and CTV motion (CoM), the PCC values showed moderate positive correlation in the RL direction (0.56), weak positive correlation in the AP direction (0.10), weak negative correlation in the SI direction (-0.20) and strong positive correlation in 3D (0.84). Statistically significant differences in motion between the ICD lead tip and CTV (CoM) were observed in the RL, AP and 3D directions, with p-values of 0.001, 0.000 and 0.005, respectively. However, no significant difference was found in the SI direction (p-value = 0.470). The Euclidean distances between ICD lead tip motion and CTV (CoM) motion in the RL, AP, SI and 3D directions were 13.0 mm, 12.1 mm, 10.5 mm and 7.4 mm, respectively. In the 3 cases with ICD lead tip motion > 7.0 mm, the motion difference to the CTV (> 2.0  mm) was greater than in cases with ICD lead tip motion < 7.0 mm.

**Fig. 5:**
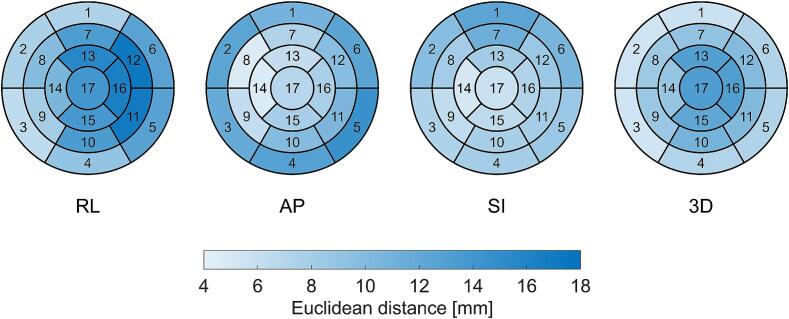
Euclidean distances between implantable cardioverter-defibrillator (ICD) lead tip motion and American Heart Association (AHA) 17 left ventricle segments motion in the right-left (RL), anterior-posterior (AP), superior-inferior (SI) and 3D directions, displayed on AHA 17-segment bull’s-eye maps.

The Euclidean distance between ICD lead tip motion and 17 LV segments motion in the RL, AP, SI and 3D directions are shown on the 17-segment bull’s-eye maps in Fig. 5. The Euclidean distances between the ICD lead tip motion and 17 segments motion were 12.0 ± 3.4 mm, 9.2 ± 2.9 mm, 8.2 ± 1.9 mm and 9.1 ± 2.6 mm in the RL, AP, SI and 3D directions, respectively. In 3D, the smallest Euclidean distance was observed in segment 3, while the largest occurred in segment 17.

## 4 Discussion

This analysis estimated cardiac motion of the CTV (CoM and individual points), the 17 LV segments and the ICD lead tip as a surrogate for STAR. With 10 datasets captured within the harmonized RAVENTA framework, this analysis provides reference data on patient- and segment-specific cardiac motion for STAR treatment planning in VT patients.

We demonstrated that the CTVs (CoM) moved 3.4 ± 1.4 mm, and 10 out of 12 CTVs exhibiting < 5 mm cardiac motion in 3D. Cardiac CTs were acquired during end-inspiratory breath-hold so that our results present an approximation to cardiac motion without respiratory motion. Similarly, Ouyang et al. [17] investigated the motion of the cardiac substructures including LV (centroids), using breath-hold cardiac-gated CT in 10 transcatheter aortic valve replacement patients, reporting that over 90 % of the motion was < 5 mm. Petzl et al. [20] used motion data from the ablation catheter and reported an average cardiac motion of 1.62 ± 1.21 mm. Li et al. [22] analyzed the VT substrate (centroid) of 12 STAR-treated patients with refractory arrhythmia using 4D cardiac CT and 4D CT with a deformation vector field, reporting a mean maximum displacement of the VT substrate in 3D due to cardiac pulsation of 5.2 mm (range: 2.6–8.0 mm). A recent study measured the displacement of cardiac devices and lead tips across the cardiac cycle using breath-hold 4D cardiac CT in 31 patients undergoing catheter ablation, revealing significant variability in cardiac contractile motion (range: 1–15 mm) [21]. Cardiac motion of the CTV (CoM) in our study generally fell within this range.

While the CoM motion of the CTV represents an averaged value, the motion of individual CTV points provides complementary cardiac motion information. The comparison of cardiac motion of the CoM for individual CTVs with the mean value of the individual CTV points suggested that cardiac motion was relatively uniform and centralized across the CTV points, indicating coherent movement with no substantial deviations in different regions of a specific CTV. Therefore, CTV (CoM) motion can serve as cardiac motion estimation, except when CTV moves in a significant regional inhomogeneous manner.

In the radiosurgery platform CyberKnife® (Accuray, Sunnyvale, USA), the tip of the ICD lead is used as a tracking marker for motion management during STAR for VT [30], [31], [32]. In our analysis, the ICD lead tip motion in 3D was 4.9 ± 2.2 mm, which is smaller than the RV lead tip motion observed in the study by Wu et al. [21] (8.6 ± 3.5 mm). For 7/10 patients, the motion difference between ICD lead tip (near segment 14) and CTV in 3D was ≤ 2 mm. In these 7 cases, the ICD lead tracking technique appears to be sufficient as a reference for cardiac motion of the CTV. It was observed that as the motion of the ICD lead tip increased, the difference in motion increased accordingly, making it less reliable to use ICD lead tracking as a reference for cardiac motion estimation. A lower value of the Euclidean distance between ICD lead tip motion and 17-segment motion indicates that the motion pattern of the specific segment is relatively more aligned with the ICD lead tip motion. The Euclidean distances were generally axisymmetric on the 17-segment model bull’s-eye maps in Fig. 5. Overall, they decreased from the apical segments to the mid-cavity and basal segments in the RL and 3D directions, while they increased from the apical segments to the mid-cavity and basal segments in the AP and SI directions. In those segments with lower Euclidean distances, ICD lead tracking seems to be an adequate surrogate for estimating cardiac motion during STAR. Segments 13 and 15–17 showed the highest Euclidean distances, suggesting their motion was least correlated with the ICD lead tip. For the 10 patients, segments 1–9 and 14 were more suitable for ICD lead tracking.

Overall, cardiac motion of the CTV and 17 LV segments showed asymmetry, similar to previously published studies [33], [34], [35]. It exhibited considerable individual variability in both the CTV and the 17 LV segments in 10 patients, which is consistent with the STOPSTORM.eu consortium review [16]. When adequate respiratory motion management strategies, such as gating, DIBH or tracking, are employed, the information on patient- and segment-specific cardiac motion is very helpful for creating an appropriate individualized cardiac ITV margin for STAR. Additionally, active motion management strategies have been under investigation in recent years [36], [37], and our motion estimation method could help identify VT patients with larger cardiac motion who would particularly benefit from them. These can greatly enhance the accuracy of radiation delivery in STAR treatment for VT. Compared to previously published cardiac motion analysis methods, our approach requires only contrast-enhanced breath-hold ECG-gated cardiac CT at end-diastolic and end-systolic phases, which are more readily available in clinical practice and has potential for future clinical use. Moreover, since cardiac motion can significantly affect the accuracy of radiation delivery, its dosimetric impact is crucial and needs further investigation.

Our study has several limitations. This proof-of-concept study included only 10 patients. A larger, more diverse VT patient sample would better evaluate the performance of cardiac motion estimation methods and further investigate the accordance between ICD lead tip motion and CTV/LV segment motion. The contrast-enhanced breath-hold ECG-gated cardiac CT used in this study included only cardiac motion and did not account for respiratory motion. Including free-breathing 4D CT could enable comprehensive estimation of both cardiac and respiratory motion, and the proposed method could be utilized in such cardiorespiratory motion studies. Additionally, the voxel-wise CT image registration in this study relies on precise CTV definition and LV myocardium contouring for accurate cardiac motion estimation. The use of an auto-contouring tool specifically tailored for STAR [38] could significantly improve motion estimation outcomes. Moreover, the VT patients had a lower left ventricular ejection fraction (LVEF), so the observed motion may not be applicable to patients with a higher LVEF.

In conclusion, the CTVs (CoM) moved 3.4 ± 1.4 mm, and the ICD lead tips moved 4.9 ± 2.2 mm. The maximum motion per patient was observed in basal (segments 1, 3, 5 and 6) and mid-cavity (segments 8, 9 and 10) regions.

The estimated cardiac motion showed considerable individual variability in the CTV and 17 LV segments across different patients, highlighting the need for individualized cardiac ITV margins and motion management strategies in STAR. The proposed cardiac motion estimation method could enhance STAR accuracy and has potential for future clinical applications in STAR. The impact of cardiac motion on the dosimetry needs further investigation.

## Declaration of competing interest

The authors declare that they have no known competing financial interests or personal relationships that could have appeared to influence the work reported in this paper.
