# Supplementary material for: Electrocardiogram-gated cardiac computed tomography-based patient- and segment-specific cardiac motion estimation method in stereotactic arrhythmia radioablation for ventricular tachycardia
Source: Phys Imaging Radiat Oncol. 2025 Jan 21;33:100700. doi: 10.1016/j.phro.2025.100700 (PMC11795074; doi:10.1016/j.phro.2025.100700)
Supplement: Supplementary Data 1 [file mmc1.pdf]

Supplementary Table S1

Cardiac motion of the clinical target volume (CTV) points, CTV center of mass (CoM) and implantable cardioverter-defibrillator (ICD) lead tip for each patient. Each patient had one CTV, with the exception of patients 2 and 9 with two CTVs. Positive values indicate motion in the left, posterior and superior directions, while negative values indicate motion in the right, anterior and inferior directions. Abbreviations: IQR = interquartile range; RL = right-left; AP = anterior-posterior; SI = superior-inferior.

CTV	Direction	Min (mm)	Max (mm)	Mean (mm)	Median (mm)	IQR (mm)	CTV (CoM) motion (mm)	ICD lead tip motion (mm)
Patient 1	RL	-1.1	1.4	0.2	0.2	0.5	0.2	0.7
	AP	-3.7	-1.2	-2.5	-2.6	0.8	-2.5	2.7
	SI	-3.0	0.5	-0.4	-0.2	0.5	-0.4	0.1
	3D	1.3	4.2	2.7	2.8	0.9	2.6	2.7
Patient 2 CTV 1	RL	-0.9	1.3	0.1	0.1	0.6	0.1	0.0
	AP	-1.5	0.0	-0.8	-0.8	0.3	-0.8	0.9
	SI	0.0	4.1	1.6	1.4	1.1	1.6	1.1
	3D	0.6	4.3	1.9	1.7	1.0	1.8	1.4
Patient 2 CTV 2	RL	-1.1	0.7	-0.2	-0.2	0.5	-0.2	0.0
	AP	-1.7	0.1	-0.6	-0.6	0.3	-0.6	0.9
	SI	-1.7	0.7	-0.4	-0.3	0.8	-0.4	1.1
	3D	0.1	1.9	1.0	0.9	0.4	0.8	1.4
Patient 3	RL	-0.3	4.6	2.4	2.5	1.0	2.4	3.6
	AP	-3.3	1.9	-0.7	-0.7	1.1	-0.7	0.5
	SI	-4.6	0.4	-1.8	-1.9	1.0	1.8	-2.6
	3D	1.4	5.5	3.4	3.4	0.9	3.1	4.4
Patient 4	RL	-2.8	0.3	-1.5	-1.6	0.9	-1.5	5.5
	AP	-4.9	-1.4	-2.9	-2.6	1.7	-2.9	-2.7
	SI	-3.7	1.1	-1.2	-1.1	1.5	-1.2	3.3
	3D	1.8	5.5	3.7	3.5	1.7	3.5	7.0
Patient 5	RL	3.3	5.9	4.6	4.6	0.6	4.6	5.8
	AP	-5.9	-0.4	-3.1	-3.3	2.6	-3.1	1.8
	SI	-4.7	4.5	0.6	1.0	3.0	0.6	0.2
	3D	4.0	8.2	6.0	6.1	1.4	5.5	6.1

Patient 6	RL	-4.1	-0.8	-2.8	-2.9	1.0	-2.8	3.0
	AP	-5.3	-1.2	-3.2	-3.1	1.4	-3.2	1.2
	SI	-1.5	1.4	0.1	0.2	0.7	0.1	-4.1
	3D	1.6	6.3	4.3	4.4	1.6	4.2	5.3
Patient 7	RL	-2.8	2.6	0.0	0.0	1.5	0.0	0.2
	AP	-5.9	0.4	-2.3	-2.0	2.0	-2.3	3.9
	SI	-5.9	2.0	-2.0	-2.1	2.0	-2.0	-2.1
	3D	1.0	8.3	3.5	3.3	2.5	3.1	4.4
Patient 8	RL	-2.5	3.8	-0.6	-0.9	1.6	-0.6	0.8
	AP	-3.6	0.9	-2.0	-2.2	1.3	-2.0	0.0
	SI	-1.2	4.7	1.0	0.7	1.6	1.0	-1.6
	3D	0.7	5.5	3.0	3.0	1.0	2.3	1.8
Patient 9 CTV 1	RL	0.9	6.1	3.0	3.0	1.9	3.0	8.1
	AP	-6.0	-1.5	-3.4	-3.2	1.5	-3.4	0.0
	SI	-4.6	0.8	-1.2	-0.9	1.6	-1.2	2.6
	3D	2.1	8.5	4.8	5.0	2.6	4.7	8.5
Patient 9 CTV 2	RL	-1.6	7.1	2.6	2.5	5.1	2.6	8.1
	AP	-7.6	-0.4	-3.2	-2.8	2.6	-3.2	0.0
	SI	-7.7	-1.1	-4.2	-3.9	2.8	-4.2	2.6
	3D	2.7	12.2	6.3	5.8	4.9	5.9	8.5
Patient 10	RL	-1.7	5.2	1.9	2.0	3.0	1.9	6.9
	AP	-3.5	0.2	-1.5	-1.4	1.1	-1.5	1.2
	SI	-4.9	0.7	-1.9	-1.7	1.3	-1.9	-1.0
	3D	1.5	7.1	3.5	3.2	1.9	3.0	7.0
